# Large language models for biological sequence analysis in infectious disease research

**DOI:** 10.1016/j.bsheal.2025.09.007

**Published:** 2025-09-19

**Authors:** Junyu Luo, Xiyang Cai, Yixue Li

**Affiliations:** a Guangzhou National Laboratory, Guangzhou International Bio Island, Guangzhou 510005, China; b GZMU-GIBH Joint School of Life Sciences, The Guangdong-Hong Kong-Macau Joint Laboratory for Cell Fate Regulation and Diseases, Guangzhou Laboratory, Guangzhou Medical University, Guangzhou 511436, China; c Key Laboratory of Systems Health Science of Zhejiang Province, School of Life Science, Hangzhou Institute for Advanced Study, University of Chinese Academy of Sciences, Hangzhou 310024, China; d School of Life Sciences and Biotechnology, Shanghai Jiao Tong University, Shanghai 200240, China; e Shanghai Institute of Nutrition and Health, Chinese Academy of Sciences, Shanghai 200030, China; f Bioland Laboratory, Guangzhou 510005, China

**Keywords:** Protein language model, Genomic language model, Multimodal model, Infectious disease

## Abstract

Large language models (LLMs) have emerged as transformative tools in infectious disease research, offering unprecedented capabilities in analyzing biological sequences. This review summarizes three primary types of biological LLMs, including protein language models, genomic language models, and multimodal models, highlighting their architectures and applications. These models are revolutionizing key areas such as pathogen identification, evolutionary surveillance, host-pathogen prediction, and therapeutic development by enabling the interpretation of complex genomic and proteomic data at an unparalleled scale. While recent advancements are remarkable, challenges persist in data quality, long-context processing, model interpretability, and biosafety considerations. Understanding the potential and limitations of LLMs is crucial for leveraging them effectively in infectious disease research while ensuring responsible development and deployment.

## Highlights

• Large language models (LLMs) transform infectious disease research by decoding complex biological sequences.
• Biological LLMs include protein, genomic, and multimodal models for diverse tasks.
• Applications span pathogen identification, evolutionary surveillance, host-pathogen prediction, and therapeutic development.
• Challenges remain in data quality, context limits, model interpretability, real-world use, and biosafety risks.

## 1 Introduction

Infectious diseases represent a major global health challenge, contributing significantly to worldwide morbidity and mortality. Pathogen transmission and evolution are governed by complex host-pathogen dynamics, environmental factors, and selective pressures including immune responses and medical interventions [1,2]. Recent outbreaks, particularly severe acute respiratory syndrome coronavirus (SARS-CoV) and severe acute respiratory syndrome coronavirus 2 (SARS-CoV-2), have demonstrated rapid pathogen adaptability, resulting in altered transmission dynamics, enhanced virulence, and immune escape capabilities. These evolutionary adaptations strain public health systems and underscore the critical need for comprehensive genomic surveillance [[3], [4], [5]].

The challenge is compounded by the unprecedented scale and complexity of biological sequence data generated during outbreaks and routine surveillance. High-throughput sequencing technologies have produced vast datasets encompassing pathogen genomes, host responses, and evolutionary trajectories across genomics, transcriptomics, and proteomics [4,6]. However, integrating such heterogeneous data remains challenging due to inconsistent formats, standards, and biological contexts. Traditional statistical and bioinformatics approaches often struggle to meet large-scale sequence analysis demands. For instance, sequence alignment methods prove inefficient for extensive datasets, while current analytical frameworks frequently overlook long-range sequence interactions [7]. This inherent limitation of traditional methods drove the search for more sophisticated computational tools capable of efficiently integrating multi-dimensional information to enhance outbreak response speed and precision [8].

Large language models (LLMs) utilizing Transformer architectures have emerged as transformative solutions to these computational challenges. By treating genomic and protein sequences as discrete token languages, LLMs effectively capture long-range dependencies and contextual relationships within biological data, analogous to natural language processing [7,9]. Biologically, long-range dependencies refer to relationships between distant sequence elements, such as regulatory elements and coding genes in genomics, or spatially proximate amino acids that are linearly distant in three-dimensional protein structures, which are crucial for understanding complex regulatory, structural, and functional mechanisms. As previous methods have faced challenges in effectively model long-range dependencies without explicit annotation, LLMs offer the ability to recognize these patterns and capture sequence context over kilobase scales [10]. Through representation learning from large-scale biological datasets, LLMs facilitate diverse downstream applications including variant effect prediction, regulatory element identification, protein structure modeling, and functional annotation [[11], [12], [13], [14]].

**Fig. 1:**
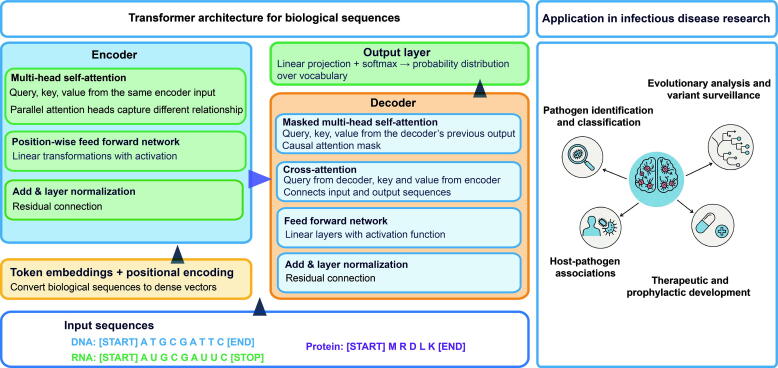
Overview of the Transformer architecture for biological sequence and its application. DNA, RNA, or protein sequences are tokenized and input into the encoder-decoder architecture. The encoder processes the input sequence by capturing contextual relationships between tokens using self-attention mechanisms and positional encoding. The decoder generates the output sequence by attending to the encoded representation. This architecture can be applied to a variety of biological tasks, including pathogen identification and classification, evolutionary analysis and variant surveillance, host-pathogen association prediction, therapeutic and prophylactic development. Some models are designed with only an encoder or only a decoder, depending on the specific task requirements. Abbreviation: DNA, deoxyribonucleic acid; RNA, ribonucleic acid; LLM, large language model.

**Fig. 2:**
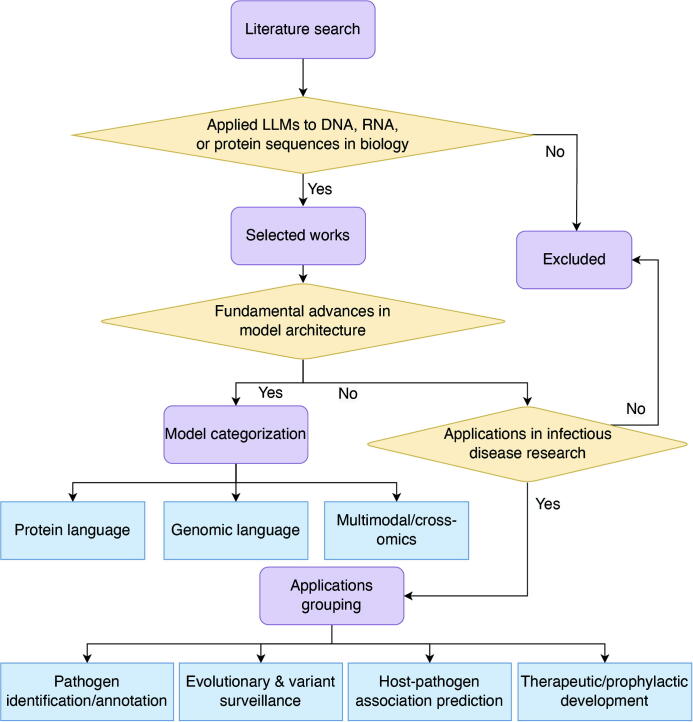
Flowchart of the literature review selection and categorization process. This flowchart illustrates the systematic process for selecting, categorizing, and summarizing literature on LLMs in infectious disease research. The process begins with a broad literature search using general and domain-specific keywords across multiple databases. A rigorous set of inclusion criteria is then applied, filtering out studies that do not focus on LLMs of biological sequences (DNA, RNA, or protein). Studies representing fundamental advances in model architecture are classified into one of three primary model types introduced in Section 2: protein language models, genomic language models, or multimodal/cross-omics models. Studies that are not architectural innovations but fall within the context of infectious disease are organized into four key domains, providing a structured overview of the application landscape in Section 3. Abbreviations: LLMs, large language models; DNA, deoxyribonucleic acid; RNA, ribonucleic acid.

In infectious disease research, LLMs demonstrate revolutionary potential across multiple domains (Fig. 1). They enable rapid analysis of large-scale pathogen genomic and proteomic data, facilitate identification and characterization of emerging variants and evolutionary dynamics, and support real-time genomic surveillance with predictive modeling of pathogen spread and adaptation [15,16]. LLMs also accelerate vaccine and therapeutic antibody design [17,18], thereby advancing the capacity to track pathogen evolution, elucidate infection mechanisms, and strengthen medical countermeasures against emerging threats. This narrative review synthesizes the rapidly evolving landscape of LLMs in infectious disease research (Fig. 2). The literature search, completed before 30th June 2025, encompassed key publications and preprints from Google Scholar, with results organized chronologically. Search terms included protein language model (n = 300), protein foundation model (n = 4), genomic language model (n = 12), deoxyribonucleic acid (DNA) language model (n = 15), ribonucleic acid (RNA) language model (n = 11), genomic foundation model (n = 10), DNA foundation model (n = 7), RNA foundation model (n = 9). Selection criteria prioritized foundational models introducing novel architectures or training paradigms (n = 33), as well as applied models demonstrating substantial performance gains in tasks directly relevant to infectious diseases (n = 17). The selected architectural designs or training paradigms were classified into three categories: protein language models (pLMs), genomic language models (gLMs), and multimodal/cross-omics models. Meanwhile, their applications were organized into four key domains: pathogen identification/annotation, evolutionary and variant surveillance, host‑pathogen association prediction, and therapeutic/prophylactic development. The aim is to provide a comprehensive overview of current advances, applications, and challenges.

## 2 Foundational models in infectious disease research

### 2.1 Basic concepts

LLMs are computational models that learn to understand and predict token sequences through self-supervised learning on massive datasets, capturing intrinsic patterns and contextual relationships [19]. Originally developed for natural language processing using Transformer architectures with self-attention mechanisms, this paradigm has been successfully extended to biology by treating biological sequences (DNA, RNA, and proteins) as linguistic entities with distinctive patterns and structural characteristics (Fig. 1). Input sequences are tokenized into basic processing units, typically individual nucleotides or k-mers for DNA/RNA sequences, and amino acids for proteins. The fundamental premise is that biological sequences, like natural language, contain inherent organizational structures and patterns that can be computationally analyzed to differentiate organisms and predict biological functions.

Early language models relied on recurrent neural networks (RNNs), long short-term memory (LSTMs), and gated recurrent units (GRUs). These models processed sequences token-by-token and captured sequential contextual information, but they struggled to model long-range dependencies due to issues like vanishing gradient. Faced with this intrinsic limitation, the Transformer architecture were developed and achieved breakthrough performance that revolutionized the field and established the foundation for modern biological language models [20]. Transformer architectures are typically structured as encoder-decoder, encoder-only, or decoder-only systems. The key innovation was the self-attention mechanism, which allows the model to weigh the importance of all tokens in a sequence simultaneously, regardless of their position. This enables efficient modeling of long-range dependencies without recurrence or convolution and facilitates parallel processing, dramatically accelerating training on large datasets [7,21].

Model development involves two key phases: pretraining and fine-tuning. During pretraining, models learn from massive genetic or protein sequence datasets, acquiring generalizable patterns, evolutionary characteristics, and structural features. Two prominent pretraining techniques are autoregressive (AR) modeling, which generates tokens sequentially based on previous predictions [22], and masked language modeling (MLM), which predicts randomly masked tokens using bidirectional context [23]. AR models excel at sequence generation, while MLM models are superior for representation learning and sequence infilling. Fine-tuning subsequently adapts pretrained models to specific tasks like viral mutation or protein function prediction, enhancing accuracy for targeted biological applications [24]. This two-stage process allows models to leverage vast unlabeled data to build a foundational understanding, which can then be specialized with much smaller, labeled datasets.

In this review, we explore three major categories of biological LLMs that have arisen from these principles: pLMs targeting amino acid sequences, gLMs designed for DNA and RNA, and multimodal models that combine diverse data types to facilitate a more comprehensive understanding (Supplementary Data).

### 2.2 Protein language models

The limitations of traditional alignment-based or early language models in capturing the full complexity of protein sequence-function relationships, especially for highly divergent proteins, have driven the development of advanced pLMs trained on massive, unlabeled protein datasets (Table 1). Several architectural frameworks have emerged, each with distinct advantages for different biological questions (Supplementary Data).

**Table 1:** Summary of protein language models described in the review.

Model	Architecture	Datasets	Context length	Parameter scale	Publish year
ESM-1b	RoBERTa Transformer (encoder-only)	UniRef50	1,024	650 M	2021
ESM-1v	RoBERTa Transformer (encoder-only)	UniRef90	1,024	650 M	2021
ProteinBert	BERT with global attention (encoder-only)	UniRef90	1,024	16 M	2021
ProtGPT2	GPT-2 Transformer (decoder-only)	UniRef50	512	738 M	2022
ProtT5	T5 (encoder-decoder)	UniRef50, BFD100	512	11 B	2022
ESM-2	Transformer with relative positional embeddings (encoder-only)	UniRef50	1,024	738 M	2023
ProGen	Transformer (decoder-only)	280 M universal sequences	512	1.2 B	2023
PoET	Transformer with sequences-of-sequences modeling (encoder-decoder)	Protein families across tens of millions of clusters	32 k	604 M	2023
xTrimoPGLM	General language model (encoder-decoder)	UniRef90, ColabFoldDB	1,024	100 B	2025

These protein language models use single-residue-level tokenization. For context length and parameter scale, if a model has multiple versions, the largest version is reported here. Abbreviations: k, represents thousands; M, millions; B, billions.

Encoder-decoder models like ProtT5 [25] and xTrimoPGLM [11] first transform protein sequences into contextual embeddings by the encoder module, then generate outputs from these representations by the decoder module. This bidirectional-to-autoregressive structure supports both understanding (e.g., alignment, classification) and generation (e.g., protein design, protein–protein interaction [PPI] prediction). While highly versatile, encoder–decoder architectures are computationally more expensive than single-module models and may require large datasets to realize their full potential.

Encoder-only models such as ESM-1b [26], ESM-1v [27], ESM-2 [28], and ProteinBert [29] focus exclusively on generating high-quality contextual embeddings without a generative decoding step. These models effectively capture residue-level dependencies through self-attention, making them suitable for secondary structure prediction and mutation effect analysis. For instance, ESM-2 enables direct inference of residue-residue contacts and three-dimensional structures via ESMFold, achieving AlphaFold2-comparable accuracy with superior computational efficiency [28]. The trade-off is that encoder-only models cannot natively generate new sequences without additional generative components.

Decoder-only models, including ProtGPT2 [30], ProGen [31], PoET [32], adopt autoregressive architectures optimized for generative tasks, synthesizing novel functional proteins through iterative amino acid prediction. Innovations like controllable generation through conditional tags and advanced tokenization have expanded their capabilities in exploring sparse protein sequence spaces [31]. However, decoder-only models have limited capacity for bidirectional context modeling, which can reduce effectiveness in certain predictive or classification tasks.

In sum, encoder-decoder models offer broad flexibility for both predictive and generative tasks, encoder-only models provide efficient and accurate embeddings for analytical and structural predictions, and decoder-only models specialize in creative sequence design. Selecting the appropriate architecture depends on the balance between understanding existing protein sequences and generating new ones, as well as computational and dataset constraints.

### 2.3 Genomic language models

gLMs are developed to understand DNA and RNA sequences through self-supervised learning on vast genomic datasets, particularly the immense length of genomes and the importance of non-coding regions (Table 2). The inherent challenge of processing ultra-long genomic sequences efficiently was the primary motivator for innovations in gLMs, leading to the development of encoder-only, decoder-only, encoder-decoder, and hybrid architectures for various tasks. Early models like DNABERT demonstrated that pre-trained bidirectional encoder representations could capture genomic syntax and semantics [33], though k-mer tokenization introduced computational inefficiencies and data leakage. Subsequent innovations were driven by the need for greater efficiency and longer context windows. DNABERT-2 [34] and GROVER [35] adopted byte pair encoding (BPE) for enhanced efficiency, while models like GPN implemented nucleotide-level tokenization for higher-resolution tasks like variant effect prediction [36]. Scaling these efforts led to foundational models like GenSLMs (trained on 110 + million prokaryotic gene sequences) [16] and Nucleotide Transformer (trained on 3,200 + human genomes and 850 diverse species genomes) [37], demonstrating strong variant prediction performance. Despite these advances, a major limitation was restricted context length (512–4,000 tokens), covering minimal genomic portions. The inability to model ultra-long-range dependencies across entire genes or regulatory regions, crucial for understanding complex genomic functions, propelled the development of new architectures to address this context length limitation. MegaDNA employs multiscale Transformer architecture for ultra-long contexts up to 96 kb in bacteriophage genomes [38]. HyenaDNA uses the Hyena operator with global convolutional filters and data-controlled gating instead of attention, enabling sub-quadratic scaling and 1 Mb context length at single-nucleotide resolution [10]. Building on StripedHyena, Evo processing 131 kb sequences through 29 Hyena layers [39]. Its successor Evo 2 represents the state-of-the-art, trained on trillions of DNA bases from 100,000 + species with 1 Mb context capability [40], using optimized StripedHyena 2 architecture for efficient extended sequence processing.

**Table 2:** Summary of genomic language models described in the review.

Model	Architecture	Datasets	Tokenization	Context length	Parameter scale	Publish year
DNABERT	BERT (encoder-only)	Human genome (Hg38.p13) sequences, 10–510 length sequences	k-mer	512	110 M	2021
GPN	ConvNet, RoFormer/Transformer, ByteNet (multiple architecture)	Arabidopsis thaliana and 7 related Brassicales species genomes	Single nucleotide	512	Not specified	2023
GenSLMs	Transformer (decoder-only)	110 M + prokaryotic gene sequences, 1.5 M SARS-CoV-2 genomes	Codon (3 nucleotides)	10,240	25 B	2023
HyenaDNA	Hyena (decoder-only)	Human reference genome	Single nucleotide	1 M	1.6 M	2023
DNABERT-2	MosaicBERT (encoder-only)	Multi-species genomes (human and other species)	BPE	4096	117 M	2024
GROVER	Transformer with next-k-mer prediction (encoder-only with dual-view structure)	5 million sequences from human genomes	BPE	512	Not specified	2024
Nucleotide Transformer	Transformer with rotary positional embeddings and GLU (encoder-only)	3,200 human genomes and 850 diverse species genomes	k-mer	12 k	2.5 B	2024
MegaDNA	Multiscale Transformer (decoder-only)	Unannotated bacteriophage genomes	Single nucleotide	96 k	145 M	2024
Evo	StripedHyena (hybrid)	2.7 M prokaryotic genomes	Single nucleotide	131 k	7 B	2024
Evo 2	StripedHyena 2 (hybrid)	128,000 + genomes across all domains of life	Single nucleotide	1 M	40 B	2025

Abbreviations: BPE, byte pair encoding; k, represents thousands; M, millions; B, billions.

A crucial point is that many gLMs, like HyenaDNA and Nucleotide Transformer, are trained on human or broad multi-species datasets. Their application to infectious disease research relies on the technique of transfer learning. By learning the fundamental “language” of DNA and RNA from vast datasets, these models acquire a foundational understanding of genomic principles that can be effectively transferred. They are then fine-tuned on smaller, pathogen-specific datasets to perform tasks like viral classification or variant effect prediction with high accuracy, often outperforming models trained only on the smaller pathogen datasets from scratch.

### 2.4 Multimodal and cross-omics models

Recognizing that biological processes are rarely governed by a single data type, and that integrating different omics data can provide a more holistic understanding, multimodal models were developed to integrate diverse data, moving beyond single-sequence analysis (Table 3). ESM-IF1 generates protein sequences folding into specified structures using AlphaFold2 predictions, trained on 12 million protein folds [41]. ESM-3, with 98 billion parameters trained on 2.78 billion proteins, jointly reasons over protein sequence, structure, and function using discrete tokenization [14]. LucaOne processes DNA, RNA, and protein data from 169,861 species, bridging sequence-based and structure-based approaches [12]. AlphaGenome predicts diverse genomic regulatory effects from DNA sequence, integrating 1 Mb context with base-pair resolution across gene expression, splicing, and chromatin accessibility modalities [42]. Its U-Net-inspired architecture combines encoder-decoder frameworks with pairwise interaction modules, enabling simultaneous 1-dimensional (D) genomic track and 2D contact map prediction through two-stage training involving pre-training and distillation. These models highlight potential in protein engineering and drug discovery, enabling controllable generation, cross-modal translation, and comprehensive biological reasoning at unprecedented scales, thereby transforming infectious disease research capabilities.

**Table 3:** Summary of multimodal models described in the review.

Model	Architecture	Datasets	Modal	Context length	Parameter scale	Publish year
ESM-IF1	GVP-Transformer with Autoregressive Decoder (hybrid)	12 M AlphaFold2 predicted protein structures	Protein sequences and structures	1,022 residues	650 M	2022
ESM-3	Transformer with Geometric Attention (encoder-decoder)	2.78 B proteins, 236 M structures	Protein sequences, structures	Variable (iterative)	98 B	2025
LucaOne	Transformer-Encoder with RoPE (encoder-only)	169,861 species (DNA, RNA, proteins)	DNA, RNA, protein sequences	Variable	1.8 B	2025
AlphaGenome	U-Net-like + Transformer blocks (other type)	ENCODE, GTEx, 4D Nucleome, FANTOM5	DNA, RNA-Seq, CAGE, PRO-cap,DNase, ATAC, histon mods, TF binding, splice sites, splite junctions, splice site usage, contact maps	1 M	Not specified	2025

Abbreviations: DNA, deoxyribonucleic acid; RNA, ribonucleic acid; BPE, byte pair encoding; k, represents thousands; M, millions; B, billions.

## 3 Application in infectious disease research

The effective control of infectious diseases requires an integrated research strategy encompassing pathogen identification, evolutionary analysis, host-pathogen interaction studies, and the development of targeted therapeutics. Timely and precise identification of pathogens forms the foundation for early outbreak detection and containment, while monitoring their evolutionary dynamics enables the early identification of novel variants, shifts in transmissibility or virulence, and supports the rational design of vaccines. Deciphering the complex molecular interactions between hosts and pathogens is critical for revealing disease mechanisms and pinpointing intervention targets, ultimately informing the development of drugs, antibodies, and vaccines that can reduce morbidity and mortality on a global scale. Emerging cases suggest that LLMs hold substantial promise for accelerating progress across these domains. By discerning sequence patterns, structural features, and functional relationships within vast biological datasets, LLMs can enhance the accuracy, efficiency, and translational impact of infectious disease research, thereby strengthening public health preparedness and response.

The choice between a pLM, gLM, or multimodal model depends on the desired resolution and the specific biological question. Early-stage, broad surveillance efforts benefit most from gLMs, whereas later-stage functional characterization and intervention design are better served by pLMs or multimodal models. For example, in viral pathogen identification, gLMs may be advantageous for detecting novel viruses because they can capture broader genomic context directly from raw sequencing reads, the type of data typically available first during an outbreak. In contrast, when the goal is to characterize known viral proteins or design therapeutics targeting specific viral components, pLMs and multimodal models are more effective. This is because they leverage learned relationships between protein structure and function, which often have a more direct connection to pathogenesis and potential drug targets.

### 3.1 Pathogen identification and annotation

Accurate and rapid pathogen identification is the cornerstone of effective disease surveillance and outbreak response. LLMs have transformed sequence-based pathogen detection and taxonomy by overcoming the limitations of slow, alignment-based methods, enabling more robust and quick identification of pathogens. PathoLM leverages pre-trained DNA foundation models like Nucleotide Transformer to enhance pathogen detection and classification [37,43]. By capturing broader genomic context with minimal fine-tuning, PathoLM outperforms traditional alignment-based methods, particularly for novel and divergent pathogens. In metagenomic applications, ViraLM detects viruses by fine-tuning DNABERT-2, surpassing existing benchmarks for identifying novel viral contigs [34,44]. This capability proved essential for early pandemic response, demonstrated through SARS-CoV-2 surveillance during the coronavirus disease 2019 (COVID-19) pandemic [45].

The challenge of accurately annotating viral proteins that lack clear sequence homology to known proteins spurred the development of pLM-based classifiers, which significantly improve viral protein annotation beyond traditional sequence homology methods. Flamholz et al. [13] developed a pLM-based classifier for prokaryotic viral proteins, capturing functional homology rather than sequence similarity alone. This enabled annotation of highly divergent viral proteins evading conventional detection. Applied to global ocean virome data, this method expanded annotated viral protein families by 29 % and identified previously uncharacterized proteins with important biological functions. The multimodal model LucaProt, incorporating sequence and structural information, further improves the accuracy of protein function prediction for RNA viruses [46,47].

### 3.2 Evolutionary analysis and variant surveillance

Understanding pathogen evolution potential immune escape mutations are crucial for predicting future threats and developing long-lasting vaccines. LLMs have substantially advanced viral evolution modeling and variant prediction. Hie et al. [48] introduced language models to viral sequences, modeling viral evolution through “grammar” (fitness) and “semantics” (antigenic change) using BiLSTM architecture. Their approach accurately identified escape mutations preserving viral function while altering immune recognition in influenza, HIV, and SARS-CoV-2, providing alignment-free methods for anticipating viral evolution and informing vaccine design. EVEscape combines SARS-CoV-2 sequences with structural information to predict viral variants before emergence, demonstrating ability to anticipate frequent mutations and consequential variants during the pandemic [49]. The co-attention Transformer model CoT2G-F bridges genotype and fitness in SARS-CoV-2, identifying immune escape mutations and forecasting fitness [15].

For evolution trajectory modeling, Evo-velocity constructs sequence similarity networks using ESM-1b embeddings, assigning directionality based on language model likelihood changes to create vector fields predicting local evolutionary direction and dynamics. This approach successfully predicted evolutionary order across timescales from viral proteins evolving over years to eukaryotic proteins evolving over geological eons, offering insights into viral immune escape and horizontal gene transfer [50]. CoVFit, trained on genotype-fitness data using ESM-2, accurately ranked unknown variant fitness and identified 959 fitness elevation events in viral evolution through late 2023 [51].

Advances in phylogenetic reconstruction also have been enhanced through models like PhyloGen [52], which leverages a pre-trained gLM to generate and optimize phylogenetic trees without dependence on traditional evolutionary models. PhyloGen demonstrates effectiveness and robustness on benchmark datasets, offering improved insights into evolutionary relationships and pathogen monitoring.

### 3.3 Host-pathogen association prediction

Identifying which hosts a pathogen can infect and understanding their interactions are critical for pandemic preparedness and therapeutic development. The substantial cost and time requirements of traditional mapping methods have led to the emergence of computational, LLM-based approaches for high-throughput prediction. PPIs are key mechanisms of pathogen infection, pathogenesis, and transmission, and thus particularly important. TUnA utilizes Transformer encoders combined with ESM-2 embeddings to predict binary PPIs and estimate prediction uncertainty, which is critical for reliability on unseen proteins [53]. The Multimodal model, LucaOne, integrating nucleic acid and protein languages, has achieved superior performance compared to existing models in predicting PPIs [12].

Predicting host range and zoonotic spillover events, a central challenge in emerging infectious disease research due to limited data, has motivated the development of LLM-based tools to address this gap. The EvoMIL framework combines ESM-1b protein embeddings to predict host species for viruses using only viral protein sequences, achieving impressive performance with area under curve (AUC) scores over 0.95 for prokaryotic hosts and 0.8–0.9 for eukaryotic hosts, while identifying key viral proteins involved in host specificity [54]. The BERT-infect model designed for viral infectivity prediction, leverages DNABERT and ViBE models on extensive nucleotide sequences to assess zoonotic spillover risk [55]. This model demonstrated enhanced performance, particularly in segmented RNA viruses, which are frequently involved in severe zoonoses but have been historically challenging to analyze due to limited data availability. It showed robust predictive capability even with partial viral sequences, making it applicable to high-throughput sequencing data and metagenomic analyses. These tools provide invaluable insights for monitoring emerging zoonotic threats.

### 3.4 Therapeutic and prophylactic development

Developing effective drugs and vaccines is a primary goal of infectious disease research, and the laborious, time-consuming nature of traditional antibody discovery, especially for hypervariable regions, has driven the adoption of LLMs to streamline and accelerate this process. The pAbT5 model [56], designed to understand and generate antibody heavy and light chain pairings, showcasing its potential for antibody design by respecting biological constraints and chain pairing preferences. EVOLVEpro combines ESM-2 with few-shot active learning for rapid in silico directed evolution of proteins [17]. It achieves functional optimization with minimal experimental data by guiding protein sequences toward desired functions while avoiding nonfunctional evolutionary dead ends. The approach has successfully optimized a therapeutically relevant monoclonal antibody against the SARS-CoV-2 spike protein. AbMAP, a transfer learning framework adapting pLMs to antibody-specific tasks, demonstrates high efficiency in antibody optimization, achieving an 82 % hit rate in refining SARS-CoV-2-binding antibodies [18]. The MAMMAL framework predicts the binding and receptor blocking activity of antibodies against influenza A hemagglutinin antigens based solely on sequence data, and demonstrated high accuracy in existing antibodies, showing potential to accelerate antibody discovery by reducing reliance on extensive laboratory testing [57].

Target-aware drug generation is exemplified by TamGen [58], a GPT-like model that generating compounds effective against specific pathogenic proteins. The aim was to overcome the limitations of traditional high-throughput screening by enabling the *de novo* design of drug candidates tailored to specific targets. This approach combines a protein encoder with a chemical language model and has successfully identified 14 compounds showing compelling inhibitory activity against the tuberculosis ClpP protease.

Resistance mechanism prediction benefits from pLMs like ProteinBERT [59], which predicts antibiotic resistance mechanisms from gene sequences with high accuracy, particularly excelling in cases with low sequence similarity to known resistance genes. The critical need to rapidly identify novel antibiotic resistance mechanisms, especially those not detectable by traditional homology-based methods, spurred the use of pLMs in this area. Such model provides interpretable predictions considering biologically relevant features such as amino acid conservation and target binding sites.

## 4 Current challenges and outlooks

Despite substantial advancements in LLMs for infectious disease research, several critical challenges must be addressed to fully realize their potential.

### 4.1 Data quality and data representation

Data quality and representation constitute fundamental barriers to training robust LLMs. Biological sequence datasets frequently contain noise, biases, and incomplete annotations that adversely impact model performance [60,61]. For example, repetitive genomic regions, comprising substantial fractions of genomes, pose particular challenges, often leading to overfitting rather than meaningful generalization. Proposed solutions include masking or down-weighting repetitive sequences and employing advanced long-range context modeling architectures [40,42].

Sampling bias represents a pervasive challenge manifesting across multiple dimensions. Natural selection bias occurs when certain pathogens are preferentially sampled due to clinical relevance or outbreak prominence, skewing datasets toward well-characterized variants[62,63]. Geographic bias emerges from uneven sequencing capacity and surveillance infrastructure, resulting in overrepresentation of specific populations or regions [45,64]. Additionally, data richness varies dramatically across pathogens, while SARS-CoV-2 has been extensively sequenced, many infectious agents lack comprehensive genomic resources [4,65,66]. This uneven landscape constrains the ability of LLMs to learn robust, biologically meaningful representations that generalize across taxa and ecological contexts. For example, a model trained predominantly on SARS-CoV-2 variants circulating in Europe and North America may fail to accurately predict the fitness or antigenicity of a novel variant emerging in an under-sampled region in Africa, as it has not learned the relevant genetic background or host-specific selective pressures.

These challenges directly impact generalizability across species and pathogens. Models trained predominantly on limited species subsets may fail to capture regulatory logic, evolutionary dynamics, and functional constraints of distantly related organisms. Although multi-species training approaches show promise [34], extraction of universal biological features remains incomplete.

Moreover, the scope and severity of these representation challenges are closely tied to the research objectives. For LLMs developed with a foundational pan-pathogen focus and aiming to generalize across a broad spectrum of infectious agents, these biases pose significant barriers to learn transferable biological insights. On the other hand, domain-specific applications targeting particular pathogens are in preference to capturing intra-species diversity and pathogen-specific evolutionary dynamics. However, although it might be beneficial from more focused and curated datasets, lack of variant-rich datasets for many pathogens still limits the model performance.

Addressing representation biases requires improved data collection and thoughtful model design. Integrating diverse and underutilized data sources, such as environmental wastewater surveillance in community [67] and traffic network [68], and experimental deep mutational screening [69], can broaden pathogen coverage and enrich biological context. Narrowing the biologically related feature space [49] and tailoring the domain-specific architectures can guide models towards biologically plausible predictions and aligned with target scenarios [61]. Promoting data sharing and collaboration across regions and disciplines is also essential to reduce accessibility gaps and ensure more balanced representation.

### 4.2 Long-context length

Long context processing enables LLMs to model interactions spanning thousands of base pairs or amino acids, improving accuracy in protein structure prediction, variant interpretation, and regulatory element identification. By integrating distant sequence information simultaneously, LLMs provide more holistic representations than traditional models limited to local contexts. However, several challenges constrain the full exploitation of ultra-long context lengths in biological LLMs.

#### 4.2.1 Computational costs and attention dilution

The primary limitation is the trade-off between performance and computational cost: self-attention mechanisms scale quadratically with sequence length, leading to increased memory usage and slower training and inference times as context windows expand. This computational burden restricts the practical maximum context length, limiting the ability to capture ultra-long-range dependencies present in biological systems.

Even with ultra-long context support, attention dilution and “lost in the middle” effects reduce effective utilization of the entire input [70,71]. In these cases, the model's attention spread too thin across all tokens as the length of the input sequence increases, and the model disproportionately focuses on sequence regions near the beginning or end of the context window, neglecting important information located centrally. This uneven attention distribution undermines the model’s capacity to fully leverage long sequences, potentially missing critical biological signals.

#### 4.2.2 Architectural and training solutions

To address these challenges, several practical strategies have emerged. Architecturally, efficient transformer variants (e.g., MegaDNA, Hyena) reduce computational complexity, enabling longer context windows without prohibitive resource demands [10,38]. Techniques such as grouped query attention (GQA) further optimize memory and compute usage by splitting attention heads into groups that share key-value vectors, effectively reducing the non-parametric costs that grow linearly with context size [72]. Sparse and structured attention patterns, as seen in models like Longformer, BigBird, and Transformer-XL, also contribute by focusing attention on relevant tokens through block-sparse or global–local attention, which mitigates the computational burden while preserving essential contextual information [73].

Training strategies that emphasize samples rich in long-range dependencies help models implicitly learn to prioritize distal interactions [74]. Additionally, prompt compression and selective context pruning techniques reduce input length by removing redundant or less relevant tokens, thereby concentrating the model’s attention on the most critical parts of the context [75,76]. Such methods not only improve efficiency but also enhance the signal-to-noise ratio in attention distributions.

### 4.3 Model interpretability and trust

A significant barrier to the clinical and public health deployment of LLMs is their “black box” nature. While they can make highly accurate predictions, understanding why a model made a particular decision is often difficult. This lack of interpretability undermines trust, as it is hard to verify whether the model is relying on genuine biological signals or spurious correlations in the training data. For example, a model predicting a variant's high fitness is far more trustworthy if it can highlight the specific mutations in the receptor-binding domain that are known to increase affinity.

To address this, researchers are adapting interpretability techniques from computer science, such as saliency maps (to highlight important input features), attention visualization, and methods like SHapley Additive exPlanations [77]. These methods help reveal which parts of a sequence (e.g., specific nucleotides or amino acids) were most influential in a model's prediction, but their findings still require validation in biological applications. Building trust requires not only improving these techniques but also validating model reasoning against established biological knowledge.

### 4.4 Validation and benchmarking

Evaluation of pathogen-related models is hindered by limited and biased datasets, with benchmarking often relying on synthetic or narrowly sampled data that fail to capture the complexity and diversity of real-world sequences [55], resulting in incomplete assessments of robustness and reliability [64]. Reported performance, frequently based on custom datasets, may not generalize, and while metrics such as perplexity, AUROC/AUPRC, and zero-shot accuracy are common, their relevance varies by task. LLMs are further challenged by sensitivity to input variations, producing variable or hallucinated outputs [78], and by domain shift, where performance degrades on novel data (e.g., emerging viral lineages), increasing false positives or negatives with potentially serious public health consequences. Addressing these risks requires robust, community-wide benchmark datasets that span diverse pathogens and temporal and geographic variation, paired with biologically relevant evaluation metrics and continuous human oversight to ensure reliable model generalization and safe application in public health contexts.

### 4.5 Translation to practice

Despite promising research, the translation of biological LLMs into real-world clinical or public health pipelines is still in its infancy. Most models remain research tools, and significant hurdles, such as the need for large, curated domain-specific datasets, rigorous external validation across diverse populations and pathogens, integration with existing clinical and public health information systems, interpretability of outputs for non-technical end users, and compliance with data privacy and regulatory requirements, must be overcome for practical deployment. Furthermore, deploying these models in real-time surveillance systems requires robust infrastructure, continuous monitoring for performance degradation, and clear protocols for acting on model predictions. While some models were used for SARS-CoV-2 surveillance, broader application across other diseases and in routine public health operations is not yet standard practice. Most initiatives are still at the proof‑of‑concept or pilot stage, lacking full incorporation into routine, real‑world practice. Bridging the gap from research to reality will require close collaboration between model developers, clinicians, public health officials, and regulatory agencies.

### 4.6 Biosafety and ethical considerations

LLM applications in infectious disease research also raise critical biosafety and ethical concerns. A major issue involves leakage risk, where models can be manipulated to generate DNA sequences resembling pathogenic organisms despite safety measures [79]. This vulnerability arises because models trained on vast genomic data can produce sequences with high similarity to harmful viruses or bacteria under carefully crafted inputs, creating dual-use risks and potentially facilitating dangerous agent synthesis.

Multiple prevention strategies are required to mitigate these risks, which includes rigorous safety alignment during model training to steer generation away from harmful sequences, robust output filtering mechanisms, and stringent access controls combined with usage monitoring to prevent malicious exploitation. Transparency in model design and continuous evaluation against curated pathogen databases are essential for detecting and blocking unsafe outputs [80]. Ethical governance requires interdisciplinary collaboration among artificial intelligence (AI) developers, biosecurity experts, and policymakers to establish frameworks that balance innovation with safety, ensuring responsible development and deployment of these powerful tools [81].

## 5 Conclusion

LLMs have demonstrated remarkable potential in advancing infectious disease research through improved sequence analysis capabilities. Their ability to capture complex biological patterns and relationships has enhanced pathogen surveillance, evolutionary tracking, host-pathogen prediction, and therapeutic development. However, realizing their full potential requires addressing critical challenges in data quality, long-context processing, model interpretability, and standardized validation. Additionally, careful consideration of biosafety and ethical implications is essential for responsible deployment. Future work could focus on improving data diversity, developing more efficient and interpretable architectures for ultra-long-range dependencies, and establishing robust safety frameworks. As these challenges are addressed, LLMs are poised to become indispensable tools in the global fight against infectious diseases.
